# Glycogenic Hepatopathy: A Case Report of an Underdiagnosed Complication of Poorly Controlled Diabetes Mellitus

**DOI:** 10.1002/ccr3.71468

**Published:** 2025-11-11

**Authors:** Fariba Haghverdilou, Sahar Behnam Roudsari, Faezeh Salahshour, Masoomeh Safaei, Parandoosh Hashemizadeh, Mohammad Rahimi, Manouchehr Nakhjavani, Alireza Esteghamati, Soghra Rabizadeh

**Affiliations:** ^1^ Endocrinology and Metabolism Research Center (EMRC) Vali‐Asr Hospital, Tehran University of Medical Sciences Tehran Iran; ^2^ Department of Cardiology Imam Khomeini Hospital Complex Tehran Iran; ^3^ Department of Radiology, Advanced Diagnostic and Interventional Radiology Research Center (ADIR) Tehran University of Medical Sciences Tehran Iran; ^4^ Department of Pathology Cancer Institute, Imam Khomeini Hospital Complex, Tehran University of Medical Sciences Tehran Iran

**Keywords:** diabetes complications, diabetes control, diabetes mellitus, glycogenic hepatopathy

## Abstract

Glycogenic hepatopathy (GH) is a rare yet important complication of poorly controlled diabetes mellitus (DM), especially type 1 diabetes mellitus (T1DM). It presents with hepatomegaly, abdominal pain and elevated liver enzymes. Here, we present a case of GH in a 14‐year‐old girl with poorly controlled T1DM.

## 1 Introduction

Glycogenic hepatopathy (GH), which is characterized by hepatomegaly and elevated liver enzymes, is a rare complication of poorly controlled DM, commonly in pediatrics and young adults with T1DM [1, 2, 3, 4].

It is an underdiagnosed condition, which can be present independently [4, 5, 6, 7] or as a component of Mauriac syndrome (MS) with other extrahepatic features of short stature, delayed puberty and cushingoid appearance [7, 8, 9, 10, 11]. The pathophysiology of GH is poorly understood. The assumed mechanism is the excessive storage of glycogen in hepatocytes, basically as a result of wide fluctuations in blood glucose and insulin levels. In many cases GH is misdiagnosed as NAFLD, a more common liver involvement in DM patients which presents with similar symptoms [1, 2, 6, 9, 12, 13]. Liver biopsy remains the main tool for distinguishing GH and NAFLD and the gold standard of diagnosis [1, 2, 3, 11, 12, 14]. Since the introduction of insulin analogs, GH is rarely seen due to the improved control of blood sugar (BS) and reduced episodes of diabetic ketoacidosis (DKA); however, it still exists. Here, we present a case of GH in a 14‐year‐old girl with poorly controlled T1DM, who presented with abdominal pain, nausea and vomiting.

## 2 Case History/Examination

A 14‐year‐old girl, a known case of T1DM diagnosed at the age of 11, was admitted to our hospital due to uncontrolled BS. She had been treated with regular and neutral protamine hagedorn (NPH) insulin. However, she discontinued the NPH insulin 2 years ago due to tremor and weakness following the injections; therefore, she has been injecting only 10 units of regular insulin once a day. She has been admitted to the hospital several times due to hyperglycemia and DKA since the diagnosis. She complained of abdominal pain, predominantly in the epigastric and right upper quadrant (RUQ) area, along with nausea and vomiting in the last 2 months.

Physical examination revealed a good general condition, with body mass index (BMI) within normal range (BMI = 19.2 kg/m^2^, weight = 45 kg, height = 1.53 m), alert, well‐oriented and with normal vital signs. BS at the time of admission was 430 mg/dL. On abdominal examination mild tenderness in RUQ and hepatomegaly with a liver span of 26 cm were detected. The examination for puberty was completely compatible with her age. No sign of cushingoid features, like truncal obesity, buffalo hump, and facial plethora was observed.

After hospital admission the insulin regimen had converted to insulin analogues, rapid‐acting (aspart) and long‐acting (glargine), for more precise BS control; however, she still had episodes of hypo and hyperglycemia. Despite alteration in insulin dosage, the patient inexplicably showed episodes of hypo‐ and hyperglycemia. Considering the lack of response to insulin dose regulation and given that the patient experienced recurrent hypoglycemic episodes on long‐acting insulin, the insulin was first tapered and then discontinued. However, the patient still experienced hypoglycemia. After the complete discontinuation of insulin, serum insulin and c‐peptide levels in one hypoglycemic episode were measured. High insulin level (insulin = 25.9 micIU/mL) and low c‐peptide level (c‐peptide = 0.9 ng/dL) were compatible with exogenous insulin injection but the patient denied taking insulin. So, psychiatric consultation was performed and it was determined that the patient had been suffering from severe anxiety and depression following her parents' divorce, which had led to non‐compliance with treatment. The patient was taking more or less insulin than the prescribed dose, leading to alternating hypoglycemia and hyperglycemia.

## 3 Differential Diagnosis, Investigations and Treatment

**FIGURE 1:**
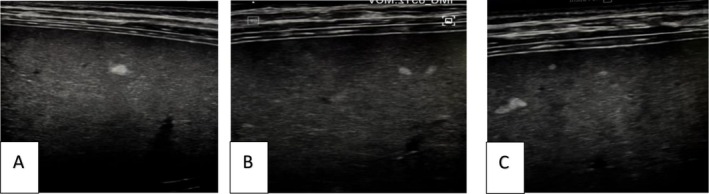
Abdominal ultrasound of the liver depicted hepatomegaly with a normal echo pattern. Several small echogenic nodules were seen during the examination of the liver with a linear probe.

**FIGURE 2:**
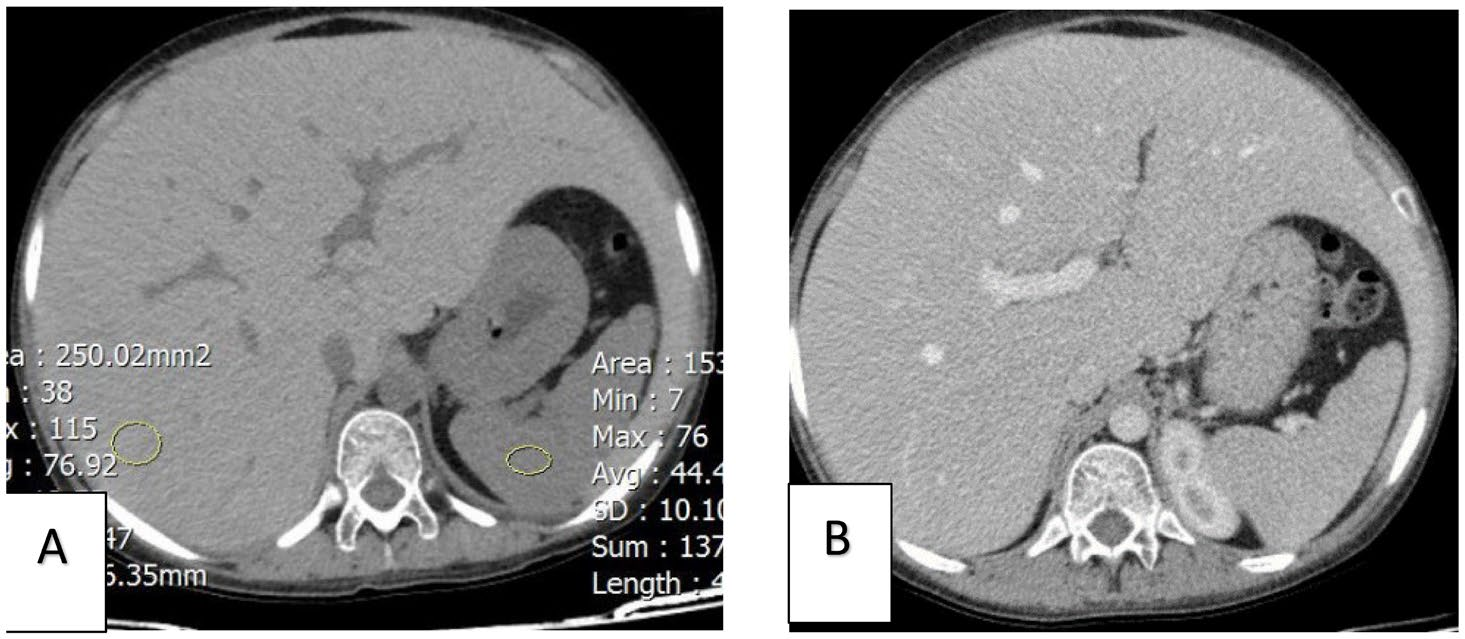
(A) Computed tomography (CT) scan of abdomen without contrast and (B) with contrast depicting hepatomegaly and diffuse increase in liver density (in non‐contrast CT scan) with no focal lesion.

Lab tests revealed anemia (Hb = 9 g/dL), HbA1C = 10.1%, elevated liver enzymes (aspartate transaminase (AST) = 499 U/L, alanine transaminase (ALT) = 280 U/L, alkaline phosphatase (ALP) = 382 U/L), and high triglyceride (TG) level (TG = 387 mg/dL). Renal and thyroid function tests were normal. Differential diagnoses for abnormal liver enzymes included: viral hepatitis, Wilson's disease, autoimmune hepatitis, hemochromatosis, celiac disease, glycogen storage diseases, alpha‐1 anti‐trypsin deficiency, drug‐induced liver injury, GH and NAFLD. Therefore, diagnostic steps were taken to explore the differential diagnoses. Workups for viral hepatitis, Wilson's disease, autoimmune hepatitis, hemochromatosis, celiac disease and human immunodeficiency virus (HIV) were negative. Abdominal ultrasound showed hepatomegaly with a normal echopattern. Several small echogenic nodules were depicted examining the liver with a linear probe (Figure 1). For more detailed examination of the nodules seen in the ultrasound, the patient went through a triphasic abdominal computed tomography (CT) scan, which revealed hepatomegaly (268 mm) and diffuse increase in liver density (hansfield = 76) with no focal lesion, similar to glycogen storage disease (Figure 2). In the CT scan, the density of the liver was not decreased; therefore fatty liver was ruled out.

**FIGURE 3:**
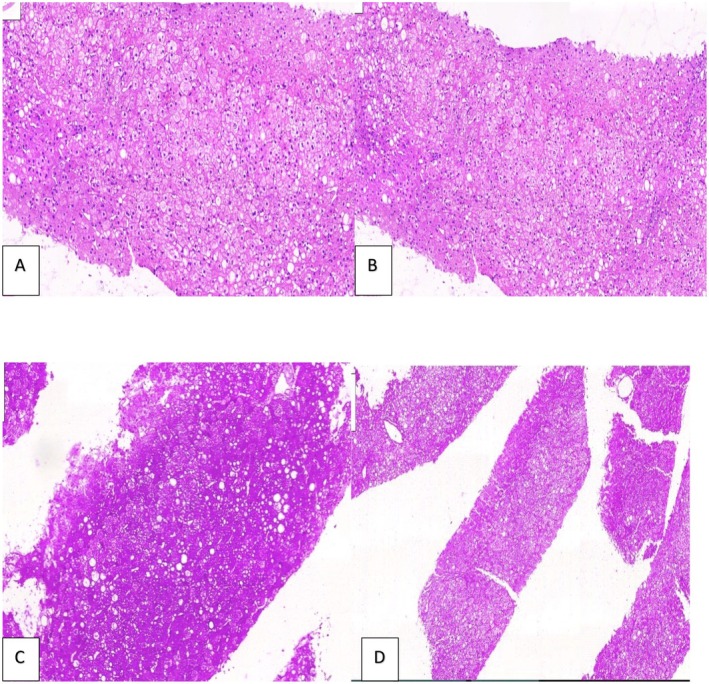
Microscopic examination shows liver tissue is composed of swollen hepatocytes with pale cytoplasm and compression of sinusoids (A, intermediate magnification. B, high magnification). The PAS stain reveals glycogen in the hepatocytes (C, intermediate magnification), which is digested by the PAS‐D stain (D, low magnification).

For the definitive diagnosis, a liver biopsy was done. According to the histopathological report, mild steatosis (predominantly microvesicular), hepatocyte swelling due to glycogen buildup, compression of sinusoid and positive periodic acid Schiff (PAS) stain were consistent with GH as a diagnosis. Diastase staining was also done and the glycogen was digested by diastase (Figure 3).

## 4 Conclusion and Results

After psychiatric consultation the patient underwent psychotherapy and after several psychiatric interviews, the patient accepted being adherent to the prescribed regimen. Finally, the plasma glucose level was controlled and after BS control liver enzymes gradually returned to normal levels within 3 weeks. Follow‐up lab tests after 3 months, including liver enzymes were within normal range and follow‐up ultrasound showed a liver span of 133 mm with no abnormal findings. All clinical symptoms including nausea, vomiting and abdominal pain were relieved. Genetic testing was not performed to rule out the diagnosis of GSD, but based on the patient's medical history, liver biopsy results, and response to treatment, including decreased liver enzymes and decreased liver size, a diagnosis of GH was made.

## 5 Discussion

We present a case of GH in a 14‐year‐old girl who presented with nausea, vomiting, abdominal pain, and elevated liver enzymes. Imaging findings were consistent with GH and biopsy confirmed the diagnosis.

GH was first introduced as a component of Mauriac syndrome (MS) in 1930 [7, 8, 9, 10, 11]. Later, it was found that GH can exclusively happen in poorly controlled DM without the other classical features of MS [1, 2, 3, 4, 5, 6, 7]. Its presentation varies, ranging from asymptomatic patients to those with abdominal pain, nausea and vomiting.

The exact mechanism of excessive glycogen storage in GH is yet to be established; however, the high glucose level and subsequent supraphysiologic doses of insulin have been proposed as the main culprit [1, 2, 6, 9, 12, 13, 14]. After food intake, the excess glucose is absorbed by hepatocytes and phosphorylated via the glucokinase enzyme, producing glucose‐6 phosphate (G6P). G6P is trapped in hepatocytes and high insulin levels, commonly administered in the setting of hyperglycemia, activate glycogen synthase enzyme, which converts G6P into glycogen, a stored form of glucose. Afterwards, the glycogen is broken down by the glycogen phosphorylase activity. The high levels of glucose in the presence of high insulin in poor glycemic control decrease glycogen phosphorylase activity, leading to further glycogen accumulation [2, 3, 15, 16, 17, 18, 19, 20].

Regarding the mechanism mentioned above it seems that with the improved glycemic control following the introduction of insulin analogues, GH is rarely seen; however, the exact incidence remains unclear [3]. Our patient used NPH and regular insulin to control her blood glucose but for unclear reasons, possibly the trauma from her parents' separation or fear of DKA, she injected insulin excessively.

In all patients with abdominal manifestations and elevated liver enzymes, other differential diagnoses including viral hepatitis, Wilson's disease, autoimmune hepatitis, hemochromatosis, glycogen storage diseases, alpha‐1 anti‐trypsin deficiency and drug‐induced liver injury should be ruled out before making a diagnosis of GH. Among all differential diagnoses, NAFLD is the most important one due to the similar clinical presentation and imaging findings. In contrast to GH, NAFLD has a poorer prognosis and the inflammatory events following steatosis might develop to non‐alcoholic steatohepatitis (NASH), cirrhosis, and hepatocellular carcinoma if left untreated [21, 22, 23]. Given the similar clinical presentation and ultrasound findings of these two entities of liver involvement in poorly controlled DM, a high level of suspicion for GH is needed in order to adopt more precise imaging modalities or biopsy. On CT scan, glycogenosis is displayed as a bright hyperdense liver in contrast to a hypodense liver seen in steatosis; however, CT scan cannot precisely differentiate between the two (Figure 2) [21]. Magnetic resonance imaging (MRI) provides more quantitative information, though it is limitedly available [24, 25, 26]. For now, liver biopsy remains the gold standard of GH diagnosis, demonstrating preserved liver tissue architecture, with no or minimal steatosis, inflammation, and fibrosis (Figure 3). Due to the glycogen content, PAS stain is positive.

Fortunately, GH is a reversible condition. Glycemic control is the mainstay of treatment and in most cases, clinical and biochemical improvements can be achieved within 2–14 weeks after glycemic control. In our patient, full remission of clinical symptoms and normalization of lab tests were achieved within 3 weeks of glycemic control [3, 14, 27, 28, 29].

## Author Contributions

**Fariba Haghverdilou:** investigation, validation, writing – review and editing. **Sahar Behnam Roudsari:** investigation, writing – original draft. **Faezeh Salahshour:** data curation. **Masoomeh Safaei:** data curation. **Parandoosh Hashemizadeh:** investigation. **Mohammad Rahimi:** investigation. **Manouchehr Nakhjavani:** supervision, validation. **Alireza Esteghamati:** supervision, validation. **Soghra Rabizadeh:** project administration, supervision, validation.

## Consent

A written informed consent (based on the patient consent policy of the Clinical Case Reports journal) was obtained from the patient.

## Conflicts of Interest

The authors declare no conflicts of interest.

## Data Availability

The data that support the findings of this study are available from the corresponding author upon reasonable request.
